# Examining psychosocial pathways to explain the link between breastfeeding practices and child behaviour in a longitudinal cohort

**DOI:** 10.1186/s12889-024-17994-0

**Published:** 2024-03-04

**Authors:** Sarah E. Turner, Leslie Roos, Nathan Nickel, Jacqueline Pei, Piushkumar J. Mandhane, Theo J. Moraes, Stuart E. Turvey, Elinor Simons, Padmaja Subbarao, Meghan B. Azad

**Affiliations:** 1 Manitoba Interdisciplinary Lactation Centre (MILC), Winnipeg, MB Canada; 2 Children’s Hospital Research Institute of Manitoba, Winnipeg, MB Canada; 3 Department of Community Health Sciences, University of Manitoba, Winnipeg, MB Canada; 4 Department of Psychology, University of Manitoba, Winnipeg, MB Canada; 5 Department of Kinesiology and the Manitoba Centre for Health Policy, University of Manitoba, Winnipeg, MB Canada; 6 Department of Educational Psychology, University of Alberta, Edmonton, AB Canada; 7 Department of Pediatrics, University of Alberta, Edmonton, AB Canada; 8 Department of Pediatrics, The Hospital for Sick Children, University of Toronto, Toronto, ON Canada; 9 Department of Pediatrics, University of British Columbia, Vancouver, BC Canada; 10 Department of Pediatrics and Child Health, University of Manitoba, Winnipeg, MB Canada; 11 Department of Pediatrics, Physiology & Dalla Lana School of Public Health, The Hospital for Sick Children, University of Toronto, Toronto, ON Canada

**Keywords:** Breast milk feeding, Breastfeeding practices, Parent-child relationship, Postpartum depression, Child behaviour

## Abstract

**Objective:**

Breastfeeding is associated with reduced postpartum depression, stronger parent-child relationships, and fewer behavioral disorders in early childhood. We tested the mediating roles of postpartum depression and parent-child relationship in the association between breastfeeding practices and child behavior.

**Study design:**

We used standardized questionnaire data from a subset of the CHILD Cohort Study (*n* = 1,573) to measure postpartum depression at 6 months, 1 year and 2 years, parent-child relationship 1 year and 2 years, and child behavior at 5 years using the Child Behavior Checklist (range 0-100). Breastfeeding practices were measured at 3 months (none, partial, some expressed, all direct at the breast), 6 months (none, partial, exclusive), 12 months, and 24 months (no, yes). Confounders included birth factors, maternal characteristics, and socioeconomic status.

**Results:**

Breast milk feeding at 3 or 6 months was associated with − 1.13 (95% CI: -2.19-0.07) to -2.14 (95% CI: -3.46, -0.81) lower (better) child behavior scores. Reduced postpartum depression at 6 months mediated between 11.5% and 16.6% of the relationship between exclusive breast milk feeding at 3 months and better child behavior scores. Together, reduced postpartum depression at 1 year and reduced parent-child dysfunction at 2 years mediated between 21.9% and 32.1% of the relationship between breastfeeding at 12 months and better child behavior scores.

**Conclusion:**

Postpartum depression and parent-child relationship quality partially mediate the relationship between breastfeeding practices and child behavior. Breastfeeding, as well as efforts to support parental mental health and parent-child relationships, may help to improve child behavior.

**Supplementary Information:**

The online version contains supplementary material available at 10.1186/s12889-024-17994-0.

## Background

Early life nutrition is critical for healthy neurodevelopment [1] and cognitive functioning [2]. Breastfeeding is the recommended source of nutrition for most infants; exclusively in the first six months of life and continuing for two years or as long as the parent and child wish to continue [3]. In Canada, 41% of infants receive exclusive breast milk up to 6 months [4]. Breastfeeding is associated with higher neurodevelopmental scores [1, 5, 6] and reduced child behavioral problems; [7, 8] however, the pathways underlying this link remain largely unknown. Studying pathways between breastfeeding practices and child behavior can inform future feeding guidelines and identify alternate factors that could be bolstered to improve child behavior.

Existing research examining the link between breastfeeding and child behavior have used inconsistent measures of breastfeeding, making results difficult to compare and often not reflective of actual breastfeeding practices. For example, many studies do not capture breast milk feeding method, yet research suggests greater protection against asthma development and improvements in working memory for children who receive breast milk directly from the breast compared to expressed breast milk [9, 10]. These differences may be due to changes in milk composition or disruptions to the parent-child bond for those who receive expressed breast milk compared to direct breastfeeding [9, 10]. To our knowledge, no research has examined breast milk feeding mode in relation to behavioral outcomes.

Two hypothesized pathways are explored in the current study to explain the link between breastfeeding practices and child behavior: (1) breastfeeding may reduce postpartum depression, and (2) breastfeeding may facilitate a positive parent-child relationship. Previous research has established a link between shorter breastfeeding duration and increased postpartum depression [11, 12], a condition that affects 18% of birthing parents in Canada in the first year after giving birth [13]. Parents who breastfeed may have improved sensitivity and greater attachment to their child, two components that can contribute to a positive parent-child relationship [14]. Increased postpartum depression is linked to lower parent-child bonding scores [15], indicating that these two mechanisms may act along the same pathway. Finally, fewer parental depressive symptoms and a strong parent-child relationship are linked to improved child outcomes, including greater executive functioning skills and behavioral regulation [16, 17]. 

These hypotheses are also guided by Attachment Theory which posits that children develop a reliance on their caregivers for security, comfort and emotional regulation [18, 19]. Breastfeeding is one behaviour that may contribute to secure attachment between the parent and child [20, 21]. Further, securely attached infants are less likely to experience behavioural problems in childhood [22]. Breastfeeding can facilitate parent-child attachment through a variety of pathways such as: (1) stimulating an oxytocin hormonal response in the parent and infant through skin-to-skin contact and nipple stimulation which produces anti-stress effects [23, 24] and (2) increasing parental sensitivity or responsiveness to their child [20]. These pathways may facilitate the development of secure attachment. At the same time, it is important to note that secure attachment develops through many pathways including responsive parenting, warm interactions, and other feeding approaches all of which are accessible and observed for families that do not breastfeed [25, 26]. 

The objectives of this paper are to (1) determine the relationship between different breastfeeding practices and child behavior at 5 years in a longitudinal cohort and to (2) determine if postpartum depression and parent-child relationship mediate the association between breastfeeding practices and child behavior.

## Methods

### Data and sample

This study uses data from the CHILD Cohort Study (www.childstudy.ca), a longitudinal Canadian pregnancy cohort, ongoing since 2008 [27]. Data from the CHILD Cohort Study were collected in four sites across Canada; Toronto, Manitoba (including participants from Winnipeg, Morden and Winkler), Edmonton and Vancouver, and included approximately 3,500 parent-child pairs. Data were collected at regular intervals starting in pregnancy; the data for the current study are from the prenatal, birth, 6 month, 1 year, 2 year and 5 year time points. Pairs were excluded if the child was born with major congenital abnormalities, a child of multiple births, children resulting from in vitro fertilization or children who would not spend at least 80% of nights in the index home. Pairs were included if the infant was born at or after 35 weeks gestation, the parent had the ability to read and write English and the pregnant parent was greater than 18 years of age (19 years of age in Vancouver) at the time of recruitment. In the current sample, infants were excluded if they had parent-reported, physician diagnosed Trisomy 21 (Down Syndrome), if they did not have data on the postnatal questionnaires or if they did not have data on the behaviour outcome, the Child Behaviour Checklist (CBCL).

Written informed consent was obtained from all participants at enrollment. The study was approved by the University of Alberta, University of British Columbia, University of Manitoba and McMaster University Human Research Ethics Boards.

### Measures

#### Breastfeeding practices

Data about breastfeeding practices were collected from birth to 24 months of age at regular time intervals using repeated questionnaires. Four measures were included: (1) breastfeeding mode at 3 months, including: no breast milk (i.e. formula feeding), combination breast milk feeding and formula feeding, breast milk only (with some direct and expressed breast milk), and breast milk only (all direct); (2) breastfeeding at 6 months, including: no breastfeeding (i.e. formula feeding), partial breastfeeding or exclusive breastfeeding, (3) breastfeeding at 12 months (no or yes), and (4) breastfeeding at 24 months (no or yes).

#### Child behavior checklist

The CBCL for ages 1 ½ − 5 years was used to assess child behavior at age 5 [28, 29]. The CBCL is a widely used 99-item parent-reported questionnaire comprised of three scales: internalizing behavior, externalizing behavior and total behavior. Internalizing behaviour comprises questions about anxiety/depression, emotional reactivity, somatic complaints, and withdrawn behaviour. Externalizing behaviour comprises questions about aggressive behaviour and attention problems. The total behaviour scale includes questions that comprise the internalizing and externalizing behaviour scales as well as sleep problems and other problems. The CBCL is standardized to a normal population (adjusted for age) and represented by a t-score with mean of 50 and SD of 10. The scales range from 0 to 100 with higher scores indicating more parent-reported behavior problems.

#### Postpartum depression

Postpartum depression was measured using the 20-item self-report Centre for Epidemiological Studies Depression Scale (CES-D) [30] at 6 months, 1 year and 2 years of age. We used the established clinical cut off (score of ≥ 16) to dichotomize the scale, identifying those at greater risk for clinically significant symptoms of depression [30]. For a full description of the CES-D see the Supplement 1.

#### Parent-child dysfunction

Parent-child relationship was measured using the self-report Parent-Child Dysfunctional Interaction subscale of the Parenting Stress Index [31] at 1 and 2 years of age. We used the established guidelines [31] to identify clinical-level parenting stress by dichotomizing the score to above and below the top 85th percentile score in the current sample (score of ≥ 20). For a full description of the Parent-Child Dysfunctional Interaction subscale see the Supplement 1.

#### Confounders

Confounders were selected based on a directed acyclic graph (Supplementary Fig. 1) [32]. Confounders in the current study include: child sex (male, female), prenatal depression (score < 16, score ≥ 16 using the self-report CES-D [30]), prenatal stress (measured using the self-report, 10-item Perceived Stress Scale [33, 34]), study site (Winnipeg, Toronto, Vancouver, Edmonton), birth mode (vaginal, cesarian), birthweight (kg), gestational age (weeks), household income ($0- $29,999, $30,000- $59,999, $60,000- $99,999, $100,000- $150,000 or over, prefer not to say), maternal education (less than post-secondary degree, post-secondary degree), maternal race (White, Asian, First Nations/Other), marital status (married/common law, single/divorced/separated/never married), smoking during pregnancy (any, none), number of older siblings (none, one, two or more), and attention deficit hyperactivity disorder genetic risk score.

### Statistical analyses

Characteristics of the current sample were described and compared to those with missing outcome data (*n* = 927). The independent relationships between breastfeeding practices and child externalizing, internalizing and total behavior scores at 5 years were tested using unadjusted and adjusted linear regression (step 1).

To establish if postpartum depression and parent-child dysfunction were potential mediating variables, we followed the Baron and Kenny approach and tested the following pathways using adjusted linear or logistic regression: the relationships between breastfeeding practices and postpartum depression and parent-child dysfunction (step 2); and the relationship between postpartum depression and parent-child dysfunction and the CBCL (step 3) [35]. All models were adjusted for the covariates listed above, including: child sex, prenatal stress, prenatal depression, study site, birth mode, birthweight, gestational age, household income, maternal education, maternal race, marital status, number of older siblings, prenatal smoking, attention deficit hyperactivity disorder genetic risk score. We present β estimates or odds ratios followed by confidence intervals for steps 2 and 3. Following this, we chose the statistically significant models to an alpha of ≤ 0.05 from both step 2 and step 3 to include in causal mediation analysis. The outcomes of interest for the mediation models included the internalizing and total behaviour for breastfeeding practices at 3, 6 and 12 months and internalizing behaviour only for breastfeeding at 24 months. These outcomes were chosen because they clearly showed an association with breastfeeding practices in step 1. We present the proportion (%) of the pathway between breastfeeding and behaviour that is accounted for by the mediator (i.e. postpartum depression, parent-child dysfunction or both). All analyses were done in R version 4.2.1 and RStudio 22.07.1, using the *mediate* package for single mediation models, and the *medflex* package for multiple mediation models. Complete case analysis was employed; participants with missing data were removed from the analysis.

## Results

### Sample description

Of the 3,296 eligible infants in the full cohort, a subsample of 2,342 infants was included in the present study (Supplemental Fig. 2). At 3 months of age, 28.3% of infants (*n* = 640) were receiving breast milk directly from the breast only, while 33.9% (*n* = 767) were receiving some expressed milk (Table 1). At 12 months, 47.5% of infants (*n* = 1,110) were still receiving some breast milk; this proportion decreased to 9.5% (*n* = 221) by 24 months. At 6 months, the prevalence of clinically high postpartum depression symptoms was 13.0% (*n* = 262 parents); and similar at 1 year (12.8%, *n* = 265) and 2 years (15.7%, *n* = 305). At 1 year, 13.6% of parents (*n* = 383) had scores ≥ 20 on the parent-child dysfunction scale, with a similar prevalence at 2 years (14.4%, *n* = 281). Mean CBCL t-scores (range from 0 to 100 with higher scores indicating more behavior problems) were 44.6 ± 9.2 for internalizing child behaviors, 39.8 ± 9.6 for externalizing behaviors, and 41.3 ± 9.2 for total behavior outcomes. Infants with missing CBCL data at 5 years were more likely to receive no breast milk at any time point, have higher postpartum depression scores at 6 months and 1 year, and be more likely to have a parent who was single/divorced/ separated or never married.

**Table 1:** Characteristics of CHILD cohort study participants included in the current analysis with and without CBCL scores at 5 years

	With CBCL Data (*n* = 2,342)				Without CBCL Data (*n* = 927)				Chi-square or Wilcoxon Test *p*-value
	n	n (%) or mean [SD]			n		n (%) or mean [SD]		
**Breastfeeding Practices**									
Breastfeeding mode 3 months	2264				756				
*No breast milk (i.e. formula only)*		281	(12.4)				151	(20.0)	< 0.001
*Breast milk and formula*		576	(25.4)				215	(28.4)	
*Breast milk only (some expressed)*		767	(33.9)				209	(27.6)	
*Breast milk only (all direct)*		640	(28.3)				181	(23.9)	
Breastfeeding at 6 months	2305				730				
*No breastfeeding (i.e. formula only)*		482	(20.9)				241	(33.0)	< 0.001
*Partial breastfeeding*		1392	(60.4)				370	(50.7)	
*Exclusive breastfeeding*		431	(18.7)				119	(16.3)	
Breastfeeding at 12 months	2335				815				
*No*		1225	(52.5)				570	(69.9)	< 0.001
*Yes*		1110	(47.5)				245	(30.0)	
Breastfeeding at 24 months	2335				815				< 0.001
*No*		2114	(90.5)				790	(96.9)	
*Yes*		221	(9.5)				25	(3.1)	
**Postpartum Depression**									
6 months	2032				521				
*Score ≥ 16*		262	(13.0)				109	(20.9)	< 0.001
1 year	2074				485				
*Score ≥ 16*		265	(12.8)				105	(21.6)	< 0.001
2 years	1941				298				
*Score ≥ 16*		305	(15.7)				49	(16.4)	0.75
**Parent-Child Dysfunction**									
1 year	2076				487				
*Score ≥ 20 (85th percentile)*		383	(13.6)				79	(16.2)	0.14
2 Years	1948				299				
*Score ≥ 20 (85th percentile)*		281	(14.4)				50	(16.7)	0.30
**Child Behaviour Checklist**									
Internalizing behaviour problems (t-score)	2342	44.6	[9.2]		-				
Externalizing behaviour problems (t-score)	2342	39.8	[9.6]		-				
Total behaviour problems (t-score)	2342	41.3	[9.2]		-				
**Confounders**									
Child sex (female)	2342	1112	(47.5)		927		434	(46.8)	0.73
ADHD genetic risk score	2051	-0.01	[1.0]		649		0.02	[1.0]	0.52
Number of older siblings	2342				926				
*None*		1239	(52.9)				521	(56.3)	0.20
*One*		804	(34.3)				290	(31.3)	
*Two or more*		299	(12.8)				115	(12.4)	
Prenatal stress (range 0–40)	2188	12.3	[6.3]		787		13.2	[6.5]	< 0.001
Prenatal depression	2180				339				
*Score ≥ 16*		339	(15.6)				167	(21.4)	< 0.001
Smoking during pregnancy	2305	157	(6.8)		860		128	(14.9)	< 0.001
Maternal education (post-secondary degree)	2288	1813	(79.2)		842		577	(68.5)	< 0.001
Maternal race	2331				882				
*White*		1739	(74.6)				602	(68.3)	< 0.001
*Asian*		369	(15.8)				137	(15.5)	
*First Nations or Other*		223	(9.6)				143	(16.2)	
Marital status (married or common law)	2298	2191	(95.3)		858		777	(90.6)	< 0.001
Household income	2289				844				
*$0- $29,999*		80	(3.5)				64	(7.6)	< 0.001
*$30,000- $59,999*		303	(13.2)				116	(13.7)	
*$60,000- $99,999*		598	(26.1)				183	(21.7)	
*$100,000- $150,000 or over*		1111	(48.5)				383	(45.4)	
*Prefer not to say*		197	(8.6)				98	(11.6)	
Birth weight (kg)	2290	3.5	[0.5]		900		3.4	[0.5]	0.07
Gestational age (weeks)	2305	39.2	[1.4]		900		39.1	[1.4]	0.04
Birth mode (vaginal)	2308	1734	(75.1)		902		667	(74.0)	0.50
Study site	2342				927				
*Winnipeg*		724	(30.9)				272	(29.3)	< 0.001
*Toronto*		499	(21.3)				274	(29.6)	
*Vancouver*		568	(24.3)				159	(17.2)	
*Edmonton*		551	(23.5)				222	(23.9)	

Notes: Chi-square test of independence is used to test differences between categorical variables. Wilcoxon Rank Sum Test is used to test differences between continuous variables. ADHD, attention deficit hyperactivity disorder. CBCL, Child Behaviour Checklist; SD, Standard Deviation; prenatal stress is measured using the Perceived Stress Scale

### Step 1: breastfeeding practices are associated with better child behavior scores

**Fig. 1:**
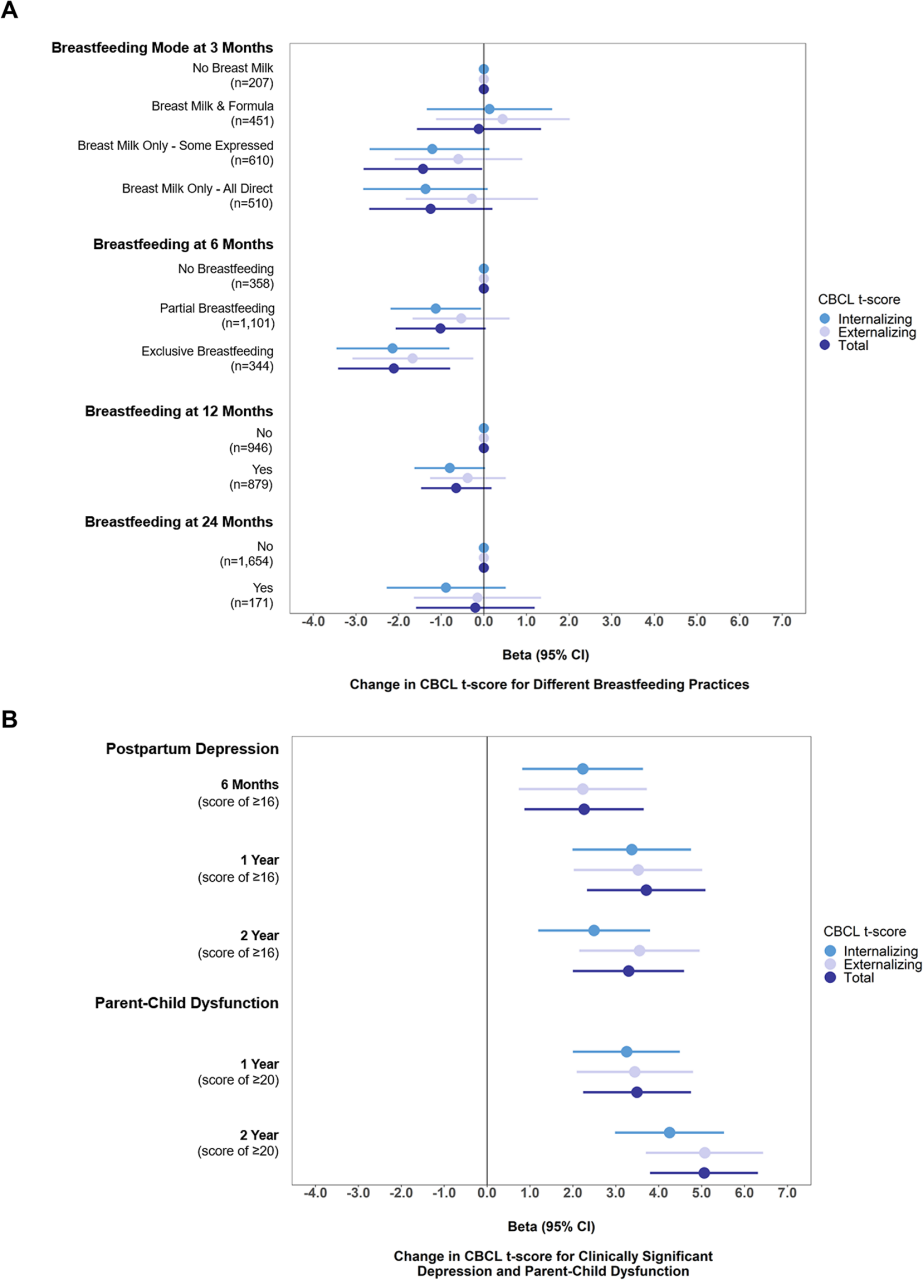
Adjusted linear regression estimates between (**A**) breastfeeding practices, (**B**) postpartum depression and parent-child dysfunction and child behaviour at 5 years in the CHILD cohort study. **Notes**: CBCL t-scores have a mean of 50 and a SD of 10. Higher scores indicate more behaviour problems. Unadjusted and adjusted β estimates, 95% confidence intervals and *p*-values for Panel A can be found in Supplementary Table (1) Adjusted β estimates, 95% confidence intervals and *p*-values for Panel B can be found in Supplementary Table (2) Depression and parent-child dysfunction scores are dichotomized to estimate clinically significant depression and relationship dysfunction. Adjusted models in all figures and tables include the following confounders: child sex, prenatal stress, prenatal depression, study site, birth mode, birthweight, gestational age, household income, maternal education, maternal race, marital status, number of older siblings, prenatal smoking, attention deficit hyperactivity disorder genetic risk score. CBCL, Child Behaviour Checklist. Reference group for breastfeeding practices is indicated by the level 0 on Panel A. Reference group for Panel B is score < 16 for depression and < 20 for parent-child relationship dysfunction

After adjusting for confounders, receiving breast milk only at 3 months (either some expressed or all direct), compared to receiving no breast milk, was related to lower internalizing and total scores (some expressed: β= -1.43; 95% CI: -2.82, -0.04; all direct: β= -1.25; 95% CI: -2.69, 0.20 for total behaviour). Exclusive breastfeeding at 6 months was related to lower internalizing scores, externalizing scores and total behavior scores (β= -2.11; 95% CI: -3.42, -0.79 for total behavior). Breastfeeding at 12 or 24 months was associated with slightly lower internalizing scores (β= -0.80; 95% CI: -1.63, 0.03 for breastfeeding to 12 months, β= -0.89; 95% CI: -2.28, 0.51 for breastfeeding to 24 months; Fig. 1A and Supplementary Table 1). Thus, direct, expressed, exclusive, and prolonged breastfeeding were all associated with better child behaviour scores, suggesting a positive and dose-dependent relationship.

### Step 2: breastfeeding practices are associated with less postpartum depression and parent-child dysfunction

Receiving breast milk only at 3 months (some expressed or all direct), compared to receiving no breast milk, was related to decreased odds of postpartum depression at 6 months (Table 2; adjusted odds ratio (OR) = 0.53; 95% CI: 0.32, 0.90, or a 47% decreased odds, and adjusted OR = 0.41; 95% CI: 0.23, 0.72, or a 59% decreased odds, respectively). Breastfeeding at 12 months was related to a decreased odds of postpartum depression at 1 year and breastfeeding at 12 or 24 months were related to decreased odds of parent-child dysfunction at 2 years **(**Table 2**)**. Direct, expressed, and prolonged breastfeeding are associated with lower postpartum depression scores and breastfeeding up to 24 months is associated with lower parent-child dysfunction scores.

**Table 2:** Adjusted logistic regression estimates between breastfeeding practices and clinically significant postpartum depression and parent-child dysfunction at 6 months, 1 year and 2 years in the CHILD cohort study

Variable	Postpartum Depression (score ≥ 16)			Parent-Child Dysfunction (score ≥ 20)		
	Time point	Sample n	Adjusted Odds Ratio (95% CI)	Time point	Sample n	Adjusted Odds Ratio (95% CI)
**Breastfeeding Mode at 3 Months** (reference: no breast milk)						
Breast milk and formula	6 Months	1573	0.71 (0.42, 1.23)	--		
Breast milk only (some expressed)	6 Months		0.53 (0.32, 0.90)*	--		
Breast milk only (all direct)	6 Months		0.41 (0.23, 0.72)**	--		
Breast milk and formula	1 Year	1594	1.07 (0.61, 1.90)	1 Year	1593	1.03 (0.62, 1.75)
Breast milk only (some expressed)	1 Year		0.81 (0.47, 1.43)	1 Year		0.77 (0.47, 1.31)
Breast milk only (all direct)	1 Year		0.60 (0.34, 1.10)^	1 Year		0.79 (0.47, 1.37)
Breast milk and formula	2 Year	1497	0.80 (0.47, 1.37)	2 Year	1503	1.18 (0.69, 2.06)
Breast milk only (some expressed)	2 Year		0.70 (0.42, 1.18)	2 Year		1.03 (0.62, 1.78)
Breast milk only (all direct)	2 Year		0.82 (0.49, 1.40)	2 Year		0.90 (0.52, 1.59)
**Breastfeeding at 6 Months** (reference: no breastfeeding)						
Partial Breastfeeding	6 Months	1598	0.80 (0.53, 1.22)	--		
Exclusive Breastfeeding	6 Months		0.70 (0.40, 1.20)	--		
Partial Breastfeeding	1 Year	1631	0.73 (0.48, 1.12)	1 Year	1630	0.87 (0.59, 1.29)
Exclusive Breastfeeding	1 Year		0.61 (0.35, 1.06)^	1 Year		0.72 (0.43, 1.19)
Partial Breastfeeding	2 Year	1532	0.86 (0.58, 1.27)	2 Year	1538	1.00 (0.68, 1.48)
Exclusive Breastfeeding	2 Year		0.76 (0.46, 1.26)	2 Year		0.76 (0.45, 1.27)
**Breastfeeding at 12 Months** (reference: No)						
Yes	6 Months		-	--		
Yes	1 Year	1632	0.69 (0.48, 0.97)*	1 Year	1631	0.85 (0.62, 1.16)
Yes	2 Year	1536	0.97 (0.70, 1.33)	2 Year	1542	0.71 (0.52, 0.97)*
**Breastfeeding at 24 Months** (reference: No)						
Yes	6 Months		-	--		
Yes	1 Year		-	1 Year		**-**
Yes	2 Year	1536	1.07 (0.65, 1.72)	2 Year	1542	0.57 (0.32, 0.96)*

Note: All estimates are odds ratios with 95% confidence intervals. Adjusted models in all figures and tables include the following confounders: child sex, prenatal stress, prenatal depression, study site, birth mode, birthweight, gestational age, household income, maternal education, maternal race, marital status, number of older siblings, prenatal smoking, attention deficit hyperactivity disorder genetic risk score. ^ *p* ≤ 0.1 (marginally significant), *p ≤ 0.05, **p ≤ 0.01, **-** models not tested because outcome occurs before exposure. -- no data available (i.e. Parent Child Dysfunction was not measured at 6 months)

### Step 3: postpartum depression and parent-child dysfunction are related to more child behavior problems

In adjusted models, postpartum depression and parent-child dysfunction were related to much higher (worse) child behavior scores (Fig. 1B; Supplementary Table 2). Postpartum depression between 6 months and 2 years were related to approximately a 0.22 to 0.37 standard deviation increase on the CBCL scales (adjusted β range from 2.23 to 3.71). Parent-child dysfunction at 1 or 2 years were related to approximately a 0.33 to 0.51 standard deviation increase in the CBCL scale (adjusted β range from 3.25 to 5.07). Both postpartum depression and parent-child dysfunction, measured up to 2 years of age, are significant predictors of higher (worse) child behaviour scores at age 5. In addition, postpartum depression at 6 months, 1 year and 2 years was associated with increased odds of parent-child dysfunction at 1 and 2 years. (Supplementary Table 3).

### Postpartum depression and parent-child dysfunction partially mediate the relationship between breastfeeding practices and child behavior

**Fig. 2:**
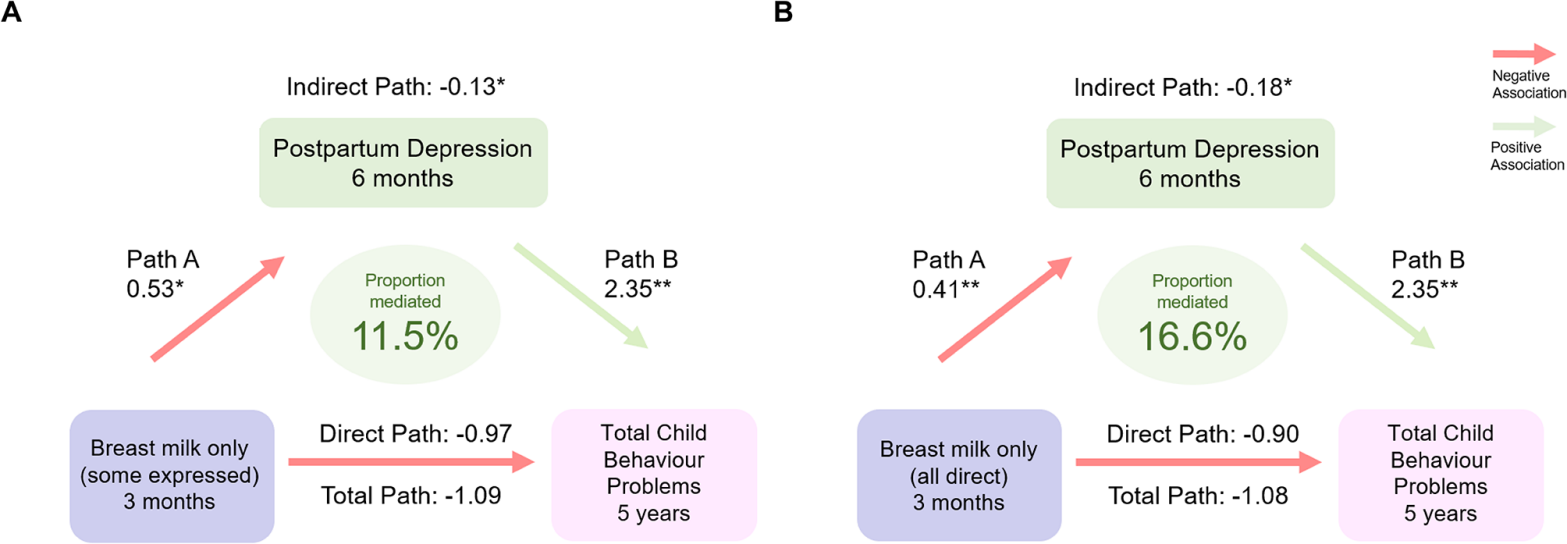
Mediation models for (**A**) some expressed breast milk and (**B**) all direct breast milk and total child behaviour problems. Notes: **Path A** are odds ratios indicating the odds of depression at 6 months (score ≥ 16) if breastfed. **Path B** are beta estimates indicating change in total behaviour problems score if depression score ≥ 16. **Total path** are beta estimates indicating the change in total behaviour problems score if breastfed. **Direct path** are beta estimates indicating the change in total behaviour problems score not through depression at 6 months. **Indirect path** are beta estimates indicating change in total behaviour problems score through depression at 6 months. Reference group for the breastfeeding practice is no breast milk at 3 months. Adjusted models in all figures and tables include the following confounders: child sex, prenatal stress, prenatal depression, study site, birth mode, birthweight, gestational age, household income, maternal education, maternal race, marital status, number of older siblings, prenatal smoking, attention deficit hyperactivity disorder genetic risk score. *p ≤ 0.05 **p ≤ 0.01

**Fig. 3:**
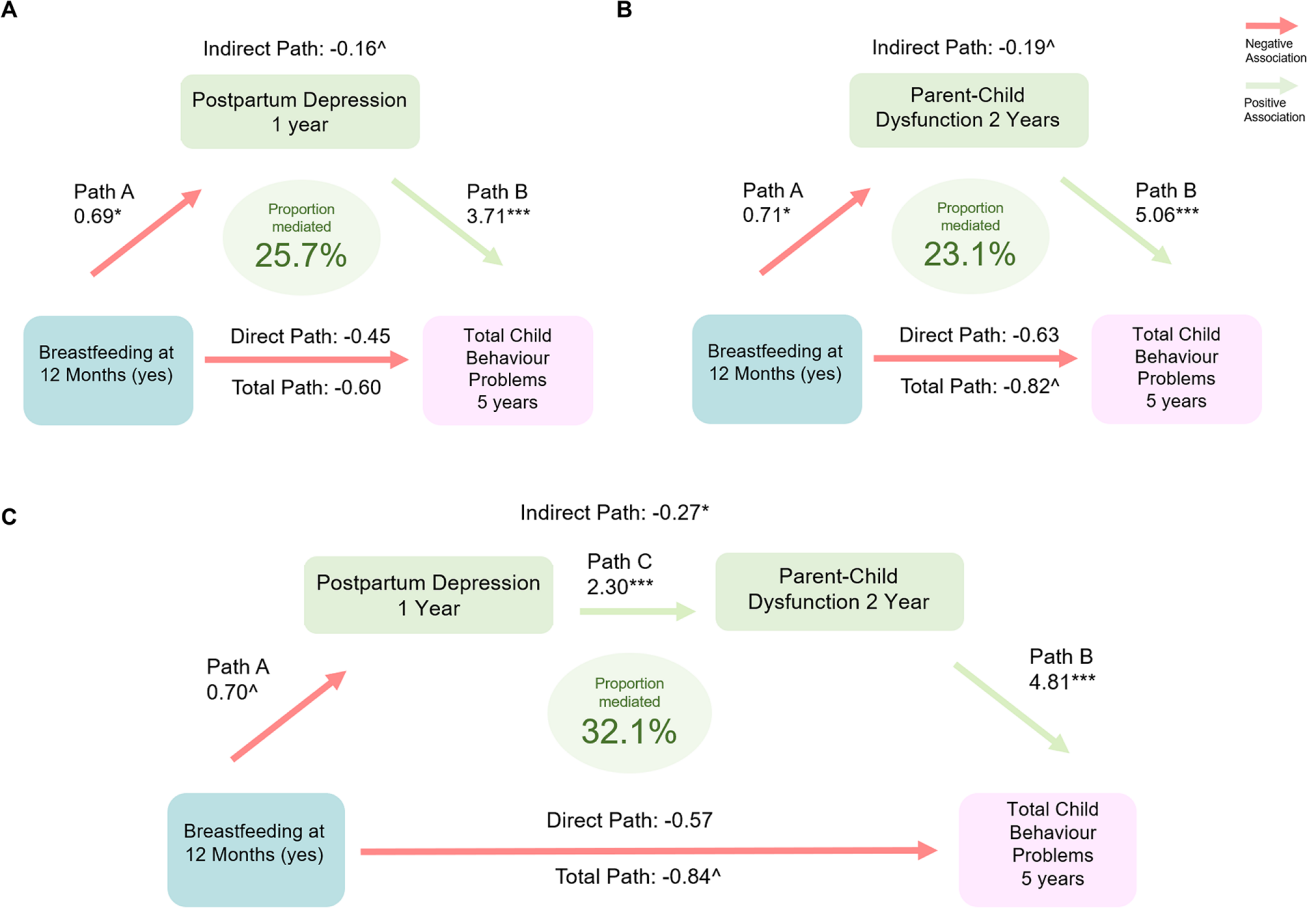
Mediation models for breastfeeding at 12 months and (**A**) postpartum depression and (**B**) parent-child dysfunction and (**C**) both mediators combined and total child behaviour problems. Notes: **Path A** are odds ratios indicating the odds of depression at 1 year (score ≥ 16) or parent-child dysfunction at 2 years (score ≥ 20) if breastfed at 12 months. **Path B** are beta estimates indicating change in total behaviour problems score if depression score ≥ 16 or parent-child dysfunction ≥ 20. **Path C** is an odds ratio indicating the odds of parent-child dysfunction score ≥ 20 if depression at 1 year score ≥ 16. **Total path** are beta estimates indicating the change in total behaviour problems score if breastfed at 12 months. **Direct path** are beta estimates indicating the change in total behaviour problems score not through depression at 1 year or parent-child dysfunction at 2 years. **Indirect path** are beta estimates indicating change in total behaviour problems score through depression at 1 year and/or parent-child dysfunction at 2 years. Reference group for the breastfeeding practice is no breastfeeding at 12 months. Adjusted models in all figures and tables include the following confounders: child sex, prenatal stress, prenatal depression, study site, birth mode, birthweight, gestational age, household income, maternal education, maternal race, marital status, number of older siblings, prenatal smoking, attention deficit hyperactivity disorder genetic risk score. ^*p* ≤ 0.1, *p ≤ 0.05 **p ≤ 0.01, ***p ≤ 0.001

In adjusted mediation models, postpartum depression at 6 months mediated 12.2% of the relationship between breast milk only (some expressed) and internalizing behavior at 5 years, and 11.5% of the relationship with total behavior scores at 5 years (Table 3; Fig. 2A). These proportions were slightly larger for breast milk only (all direct), with postpartum depression at 6 months mediating 14.2% of the relationship with internalizing behavior, and 16.6% of the relationship with total behavior scores (Table 3; Fig. 2B). Even stronger mediation was observed for postpartum depression at 1 year, which explained 15.3% and 25.7% of the relationship between breastfeeding at 12 months and internalizing behavior and total behavior scores, respectively (Table 3; Fig. 3A). Parent-child dysfunction at 2 years explained 14.9% and 23.1% of the associations between breastfeeding at 12 months and internalizing and total behavior scores, respectively (Table 3; Fig. 3B), and 31.3% of the association between breastfeeding at 24 months and internalizing behaviour. Finally, in multiple mediation, together, postpartum depression at 1 year and parent-child dysfunction at 2 years significantly mediated 21.9% and 32.1% of the relationships between breastfeeding at 12 months and child internalizing and total behavior scores at 5 years, respectively (Table 3; Fig. 3C). Postpartum depression and parent-child dysfunction are both significant mediators in the pathways between breastfeeding and child behaviour.

**Table 3:** Adjusted mediation of postpartum depression and parent-child dysfunction in the relationship between breastfeeding and child behaviour at 5 Years in the CHILD cohort study

Breastfeeding Measure	Mediator(s)	Mediation Effects	Internalizing Behaviour β (95% CI)	Total Behaviour ¥ β (95% CI)
	**Single Mediation**			
Breast milk only (some expressed) 3 Months (model *n* = 1,573)	Depression 6 Months	Total	-1.03 (-2.71, 0.59)	-1.09 (-2.77, 0.52)
		Direct	-0.90 (-2.52, 0.73)	-0.97 (-2.62, 0.64)
		Indirect	-0.13 (-0.35, -0.01)*	-0.13 (-0.35, -0.01)*
		% Mediated	**12.2%**	**11.5%**
Breast milk only (all direct) 3 Months (model *n* = 1,573)	Depression 6 Months	Total	-1.30 (-3.08, 0.22)^	-1.08 (-2.75, 0.60)
		Direct	-1.12 (-2.86, 0.38)	-0.90 (-2.54, 0.85)
		Indirect	-0.17 (-0.37, -0.02)*	-0.18 (-0.44, -0.02)*
		% Mediated	**14.2%**	**16.6%**
Breastfeeding at 12 Months (Yes) (model *n* = 1,632)	Depression 1 Year	Total	-0.71 (-1.57, 0.12)^	-0.60 (-1.43, 0.27)
		Direct	-0.60 (-1.46, 0.24)	-0.45 (-1.33, 0.38)
		Indirect	-0.11 (-0.23, -0.01)*	-0.16 (-0.28, 0.02)^
		% Mediated	15.3%	25.7%
Breastfeeding at 12 Months (Yes) (model *n* = 1,542)	Parent -Child Dysfunction 2 Years	Total	-1.05 (-1.93, -0.23)*	-0.82 (-1.70, 0.04)^
		Direct	-0.90 (-1.74, -0.03)*	-0.63 (-1.46, 0.21)
		Indirect	-0.16, (-0.34, 0.02)^	-0.19 (-0.40, 0.02)^
		% Mediated	14.9%	23.1%
Breastfeeding at 24 Months (Yes) (model *n* = 1,542)	Parent -Child Dysfunction 2 Years	Total	-0.74 (-2.15, 0.67)	-
		Direct	-0.51 (-1.92, 0.86)	-
		Indirect	-0.23, (-0.47, -0.01)*	-
		% Mediated	**31.3%**	-
	**Multiple Mediation**			
Breastfeeding at 12 Months (Yes) (model *n* = 1,436)	Depression 1 Year & Parent -Child Dysfunction 2 Years	Total	-1.05 (-1.95, -0.15) *	-0.84 (-1.75, 0.06)^
		Direct	-0.82 (-1.70, 0.06)^	-0.57 (-1.44, 0.30)
		Indirect	-0.23 (-0.42, -0.05)*	-0.27 (-0.48, -0.07)*
		% Mediated	**21.9%**	**32.1%**

Notes: Combinations of breastfeeding-mediator pairs were based on significant associations in Fig. 1 (B) and Table 2. Depression and parent-child dysfunction are dichotomized to estimate clinically significant depression (score ≥ 16) and relationship dysfunction (score ≥ 20). Adjusted models in all figures and tables include the following confounders: child sex, prenatal stress, prenatal depression, study site, birth mode, birthweight, gestational age, household income, maternal education, maternal race, marital status, number of older siblings, prenatal smoking, attention deficit hyperactivity disorder genetic risk score. ^ *p* ≤ 0.1 (marginally significant), *p ≤ 0.05, **≤0.01, - mediation not tested due to very small total effect size. ¥ Total behaviour score mediation models are represented in Figs. 2 and 3

## Discussion

In this analysis of parent-child pairs from the Canadian CHILD cohort study, we show that breastfeeding practices (including some expressed or all direct breast milk feeding) are related to better child behavior at 5 years, and that this link is partially explained by reduced postpartum depression scores and reduced parent-child dysfunction. Specifically, reduced postpartum depression scores at 6 months partially mediated the relationship between exclusive breast milk feeding at 3 months (either some expressed or all direct) and fewer child internalizing and total behavior problems. In addition, reduced postpartum depression at 1 year and reduced parent-child dysfunction at 2 years may act along the same pathway and partially mediate the relationship between breastfeeding at 12 months and fewer child internalizing and total behavior problems. These findings suggest that exclusive breastmilk feeding (i.e. either at the breast or expressed milk) in early infancy and continued breastfeeding for 12 months or longer may reduce behavior problems later in childhood. Further, they suggest that positive parental mental health and healthy parent-child relationships are key mediators in this association and represent additional target areas to help prevent child behavior problems.

### Breastfeeding practices are related to reduced child behavior problems, postpartum depression and parent-child dysfunction in the CHILD cohort study

In the current analysis, several different breastfeeding practices measured at 3, 6 and 12 months were associated with better internalizing and total behavior scores. These results align with Park (2014) who found that receiving any breast milk was more strongly related to better internalizing and total behavior scores than externalizing scores, after adjusting for sociodemographic confounders, child IQ and maternal IQ [36]. Notably, we found nearly identical numerical associations with child behavior among those who were exclusively breastmilk fed, regardless of whether they received some expressed or all direct breast milk. This result is novel as this is the first study to examine breast milk feeding mode in relation to child behavior. One previous study examined feeding mode in relation to working memory, inhibition and cognitive ability, finding significant associations with all direct breast milk, but non-significant associations with expressed breast milk for working memory only [10]. This suggests that different mechanisms underly the associations between breast milk feeding modes and memory, but these may not apply to the outcome of child behavior, inhibition or cognitive ability. Testing the differences between all direct and expressed breast milk on child behaviour with our data would require detailed quantitative information on expressed milk feeding, which was unavailable in our study. 

We did not observe a statistically significant relationship between breastfeeding at 24 months and child behaviour; however, we were underpowered for this analysis because relatively few dyads (just 9.5%) were still breastfeeding at this time. Notably, the effect estimate for internalizing behaviour was similar to other measures of breastfeeding where a significant association was observed (β= -0.89 for breastfeeding at 24 months compared to β= -1.13 for partial breastfeeding at 6 months) and, therefore, may still represent a clinically meaningful association. To our knowledge, this is the first reported analysis investigating breastfeeding at 24 months in relation to the CBCL.

Feeding exclusive breast milk at 3 months, whether expressed or directly from the breast, was related to lower postpartum depression scores at 6 months; however, the effect estimate was stronger for parents who provided direct breast milk only. It is hypothesized that direct breastfeeding infers greater benefits to the parent-child dyad compared to expressed breast milk feeding by ensuring skin-to-skin contact which produces the hormone oxytocin in the parent and baby. Higher plasma oxytocin concentrations are related to reduced postpartum depression symptoms [37] and as well as improved parent- infant interaction [38], the two mechanisms examined here. It is also important to acknowledge the potential of a reverse causal relationship whereby those with postpartum depression were less likely to breastfeed. In a previous study, depression measured at 1 week postpartum was related to breastfeeding cessation and/or lower breastfeeding self-efficacy scores at 8 weeks [39]. In the current sample, we do not know the exact timing of depression onset; however, in all models, we were able to adjust for prenatal depression, a known predictor of postnatal depression and breastfeeding duration [40, 41]. 

Breastfeeding at 12 months and 24 months were related to reduced parent-child dysfunction at 2 years. It has been shown that parents who breastfeed spend more time in close interactive behaviors with their infants [42] and express higher sensitivity to their infants [14]. Sensitivity enhances the parent-child bond, thus, longer breastfeeding duration may allow more time for sensitivity, and perhaps bonding, to develop. This may explain why breastfeeding at 12 and 24 months, but not breastfeeding at the earlier time points, is related to lower parent-child dysfunction. However, a reverse pathway is also possible, where more challenging infant temperament may disrupt the parent-child relationship [43] as well as the continuation of breastfeeding to 24 months.

Our study demonstrates that postpartum depression and parent-child dysfunction between 6 months and 2 years were related to more child behavior problems at 5 years. One mechanism that may explain these links, which have been observed in other studies [16, 44, 45], is dysregulation of the hypothalamic–pituitary–adrenal axis and cortisol secretions [46]. Early life stress, such as exposure to postpartum depression or parent-child dysfunction, may be related to an increased cortisol response, which can alter brain structure and neurodevelopmental functioning [47, 48]. Other possible mechanisms include shared genetic risk for emotional dysregulation [49], reduced home learning stimulation for children of parents with depression [44], or other environmental stressors that may affect the mental health and behavioral regulation of both the parent and/or the child.

### Postpartum depression and parent-child dysfunction partially mediate the association between breastfeeding practices and child behavior

We identified significant mediation pathways between different breastfeeding practices and child behavior at 5 years. Our results align with one previous study showing that responsive parenting partially mediates the relationship between breastfeeding duration and child internalizing behavior [50]. In the current study, high depression scores at 6 months partially explained the pathway between breast milk only (some expressed or all direct) at 3 months and child internalizing and total behavior problems at 5 years. The direct and indirect pathways have small effect sizes (between 0.01 and 0.11 of a standard deviation in behavior scores) but can still be interpreted as clinically meaningful when extrapolated to the population level because infant feeding is a universal exposure. This is explained by Rose’s Theorem, which posits that a large number of people at a small risk may result in more incidence of the outcome than the small number who are at a high risk [51]. In an unadjusted number needed to treat analysis, 23 (95% CI: 13.1, 73.1) infants would need to be directly breastfed, compared to formula fed at 3 months, for one additional child to have a total behavior score that is 1 SD (or 10 points) above the normative population mean of 50.

Combining postpartum depression at 1 year and parent-child dysfunction at 2 years in the same mediation model yielded the largest proportion mediated and indirect effect size for total behaviour problems, indicating that accounting for both mediators together does the best job of explaining the link between breastfeeding at 12 months and total child behavior scores. However, these two mediators do not fully account for this relationship. Other potential mediators could include (1) components of breast milk such as fatty acids or human milk oligosaccharides [52, 53], which have been shown to influence neurodevelopment, and (2) the composition of the gut microbiome, which is shaped by breast milk feeding and may be related to child behavior through the gut-brain axis [54]. 

### Strengths and limitations

This is the first study to examine how different breastfeeding practices (including expressed or direct breast milk feeding and breastfeeding duration to 24 months) are related to child behavior problems at age 5 years using a large longitudinal cohort with detailed assessments of early life exposures. This study is limited by self or parent-reported depressive symptoms, parent-child dysfunction and child behavior scores, introducing self-report bias to the data. Previous studies have shown that parental depressed mood is related to increased parent-reported child behaviour problems [55, 56], however, an older study indicated that this bias is minimal [57]. In addition, this limitation is countered by the fact that those with missing data on behavior scores (and therefore excluded from the present study) are also more likely to have higher depression scores, and be receiving no breast milk at 3 months. Our sample also has a higher socioeconomic status than the Canadian general population and better CBCL scores than the normative United States sample used to derive clinical CBCL cut offs. In the current sample, only 1.6% of children met the clinical cut off (≥ 65) for total behavior problems, which represents approximately the top 10% in the normative sample [29]. Thus, those with the most vulnerability are underrepresented in this sample, perhaps underestimating the true relationship between breastfeeding practices, depression and behavior problems. We also lack data on the proportion of expressed milk or formula consumed. Complete case analysis was used in this study; therefore, the sample size differs slightly between models. Finally, unmeasured residual confounding, such as infant health problems, birth or postpartum complications, and measures of parenting style or the home environment, may be possible explanations for the observed results.

## Conclusion

Our study demonstrates a positive relationship between breastfeeding practices and improved child behavior scores at five years, and this relationship is partially mediated through a reduction in postpartum depression scores and parent-child dysfunction. We observed similar numerical associations for direct and expressed breast milk feeding, providing evidence to support breastfeeding in all forms as a possible protective factor against the development of child behavior problems. Our findings further suggest that, beyond breastfeeding, services that directly address postpartum depression and positive parenting could support positive child behavior. Future work should examine other mediating pathways to more comprehensively understand the multifaceted roles of breast milk and breastfeeding on child neurodevelopment.

### Electronic supplementary material

Below is the link to the electronic supplementary material.

Supplementary Material 1

## Data Availability

A list of variables available in the CHILD Cohort Study is available at https://childstudy.ca/for-researchers/study-data/. Researchers interested in collaborating on a project and accessing CHILD Cohort Study data should contact the Study’s National Coordinating Centre (NCC) to discuss their needs before initiating a formal request. To contact the NCC, please email child@mcmaster.ca. More information about data access for the CHILD Cohort Study can be found at https://childstudy.ca/for-researchers/data-access/.

## Abbreviations

CBCL: Child Behaviour Checklist
OR: Odds ratio
SD: Standard deviation
CES-D: Centre for Epidemiological Studies Depression Scale
