# Correction to: Examining psychosocial pathways to explain the link between breastfeeding practices and child behaviour in a longitudinal cohort

**DOI:** 10.1186/s12889-024-19043-2

**Published:** 2024-06-17

**Authors:** Sarah E. Turner, Leslie Roos, Nathan Nickel, Jacqueline Pei, Piushkumar J. Mandhane, Theo J. Moraes, Stuart E. Turvey, Elinor Simons, Padmaja Subbarao, Meghan B. Azad

**Affiliations:** 1 Manitoba Interdisciplinary Lactation Centre (MILC), Winnipeg, MB Canada; 2 Children’s Hospital Research Institute of Manitoba, Winnipeg, MB Canada; 3 Department of Community Health Sciences, University of Manitoba, Winnipeg, MB Canada; 4 Department of Psychology, University of Manitoba, Winnipeg, MB Canada; 5 Department of Kinesiology and the Manitoba Centre for Health Policy, University of Manitoba, Winnipeg, MB Canada; 6 Department of Educational Psychology, University of Alberta, Edmonton, AB Canada; 7 Department of Pediatrics, University of Alberta, Edmonton, AB Canada; 8 Department of Pediatrics, The Hospital for Sick Children, University of Toronto, Toronto, ON Canada; 9 Department of Pediatrics, University of British Columbia, Vancouver, BC Canada; 10 Department of Pediatrics and Child Health, University of Manitoba, Winnipeg, MB Canada; 11 Department of Pediatrics, Physiology & Dalla Lana School of Public Health, The Hospital for Sick Children, University of Toronto, Toronto, ON Canada

**Correction to: BMC Public Health 24, 675 (2024)**

10.1186/s12889-024-17994-0

In the original publication of this article were 3 value errors in the results, discussion and Table 3 respectively. The incorrect and correct information is listed in this correction article, the full Table 3 can be accessed via the original article. These corrections do not change the main conclusions of the paper and do not result in any changes to the interpretation of the study results.

## Incorrect

### Results section

Even stronger mediation was observed for postpartum depression at 1 year, which explained 79.7% and 25.7% of the relationship between breastfeeding at 12 months and internalizing behavior and total behavior scores, respectively (Table 3; Fig. 3A).

### Discussion section

The direct and indirect pathways have small effect sizes (between 0.01 and 0.23 of a standard deviation in behavior scores) but can still be interpreted as clinically meaningful when extrapolated to the population level because infant feeding is a universal exposure.

**Table 1:** **Table 3**

Breastfeeding Measure	Mediator(s)	Mediation Effects	Internalizing Behaviour β (95% CI)	Total Behaviour ¥ β (95% CI)
Breastfeeding at 12 Months (Yes) (model *n* = 1,632)	Depression 1 Year	Total	-2.94 (-6.96, 0.73)	-0.60 (-1.43, 0.27)
		Direct	-0.60 (-1.46, 0.24)	-0.45 (-1.33, 0.38)
		Indirect	-2.34 (-5.54, 0.50)^	-0.16 (-0.28, 0.02)^
		% Mediated	79.7%	25.7%

## Correct

### Results section

Even stronger mediation was observed for postpartum depression at 1 year, which explained 15.3% and **25.7%** of the relationship between breastfeeding at 12 months and internalizing behavior and total behavior scores, respectively (Table 3; Fig. 3A).

### Discussion section

The direct and indirect pathways have small effect sizes (between 0.01 and **0.11** of a standard deviation in behavior scores) but can still be interpreted as clinically meaningful when extrapolated to the population level because infant feeding is a universal exposure.

**Table 2:** **Table 3**

Breastfeeding Measure	Mediator(s)	Mediation Effects	Internalizing Behaviour β (95% CI)	Total Behaviour ¥ β (95% CI)
Breastfeeding at 12 Months (Yes) (model *n* = 1,632)	Depression 1 Year	Total	-0.71 (-1.57, 0.12)^	-0.60 (-1.43, 0.27)
		Direct	-0.60 (-1.46, 0.24)	-0.45 (-1.33, 0.38)
		Indirect	-0.11 (-0.23, -0.01)*	-0.16 (-0.28, 0.02)^
		% Mediated	15.3%	25.7%

