# Supplementary material for: Examining psychosocial pathways to explain the link between breastfeeding practices and child behaviour in a longitudinal cohort
Source: BMC Public Health. 2024 Mar 4;24:675. doi: 10.1186/s12889-024-17994-0 (PMC10910759; doi:10.1186/s12889-024-17994-0)
Supplement: Supplementary file 1 — Supplementary Material 1 [file 12889_2024_17994_MOESM1_ESM.pdf]

Supplementary Methods

Postpartum Depression

The Center for Epidemiological Studies Depression Scale is designed to measure depressive symptoms in the general population and includes items that assess current depressed mood, feelings of guilt, worthlessness and hopelessness, loss of appetite and sleep disturbance. Each question is scored from 0 to 3 and summed together to create a continuous measure, with higher scores indicating more depressive symptoms. The scale ranges from 0 to 60.

Parent-Child Dysfunction

The Parent-Child Dysfunctional Interaction subscale includes 12 questions, scored from 1 to 5, that measure parent satisfaction with their interactions with their child and may indicate the strength of the parent-child bond. The scale ranges from 12 to 60 with higher scores indicating more parenting stress and dysfunctional interaction between the parent and child.

Supplementary Tables and Figures

Supplementary Figure 1: Directed Acyclic Graph Displaying Possible Mediation and Confounder Pathways in the Relationship between Breastfeeding Practices and Child Behaviour at 5 Years in the CHILD Cohort Study

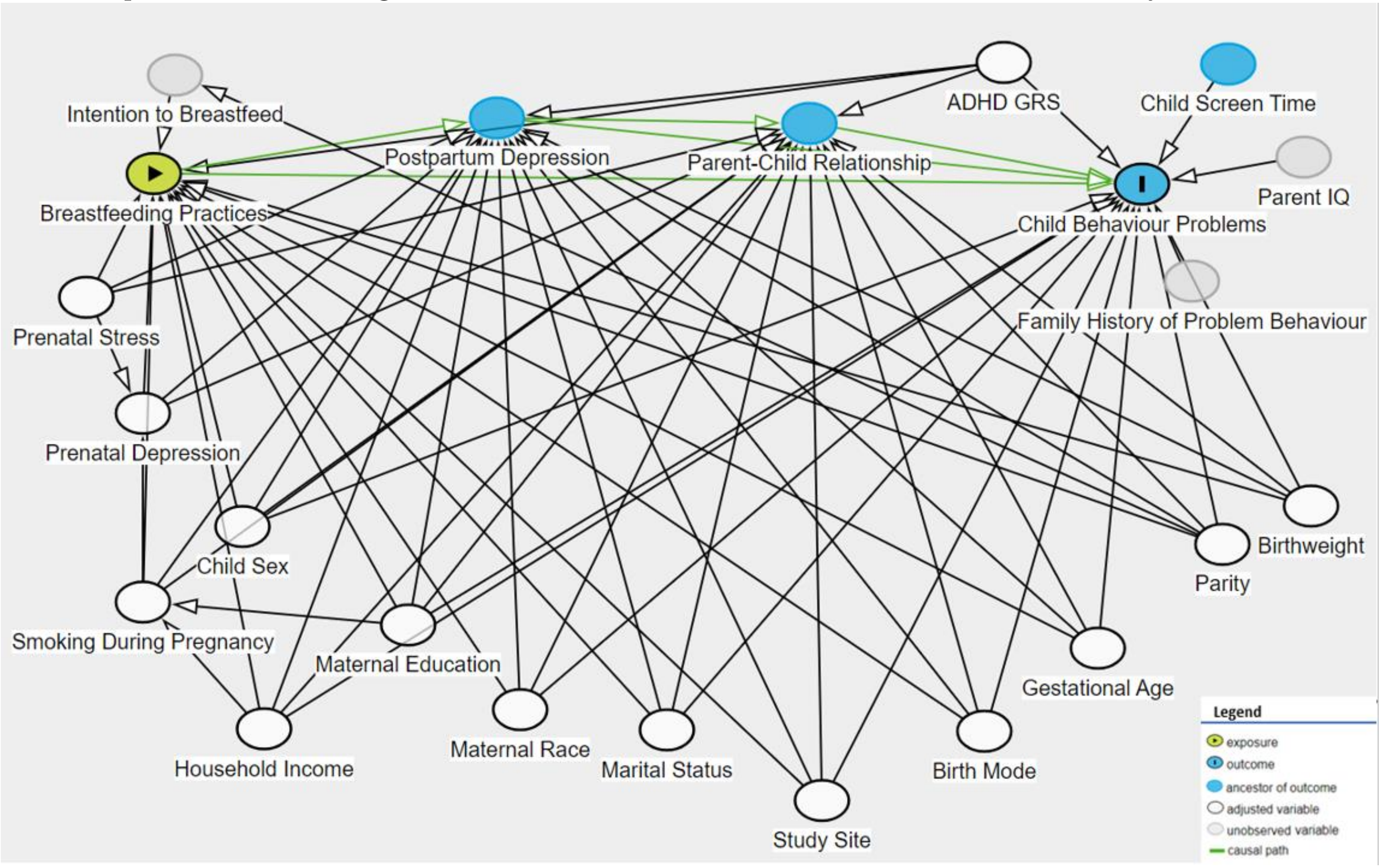

Notes: ADHD GRS, Attention Deficit Hyperactivity Disorder Genetic Risk Score

Supplementary Figure 2: CONSORT Flow Diagram for the Present Analysis of the CHILD Cohort Study

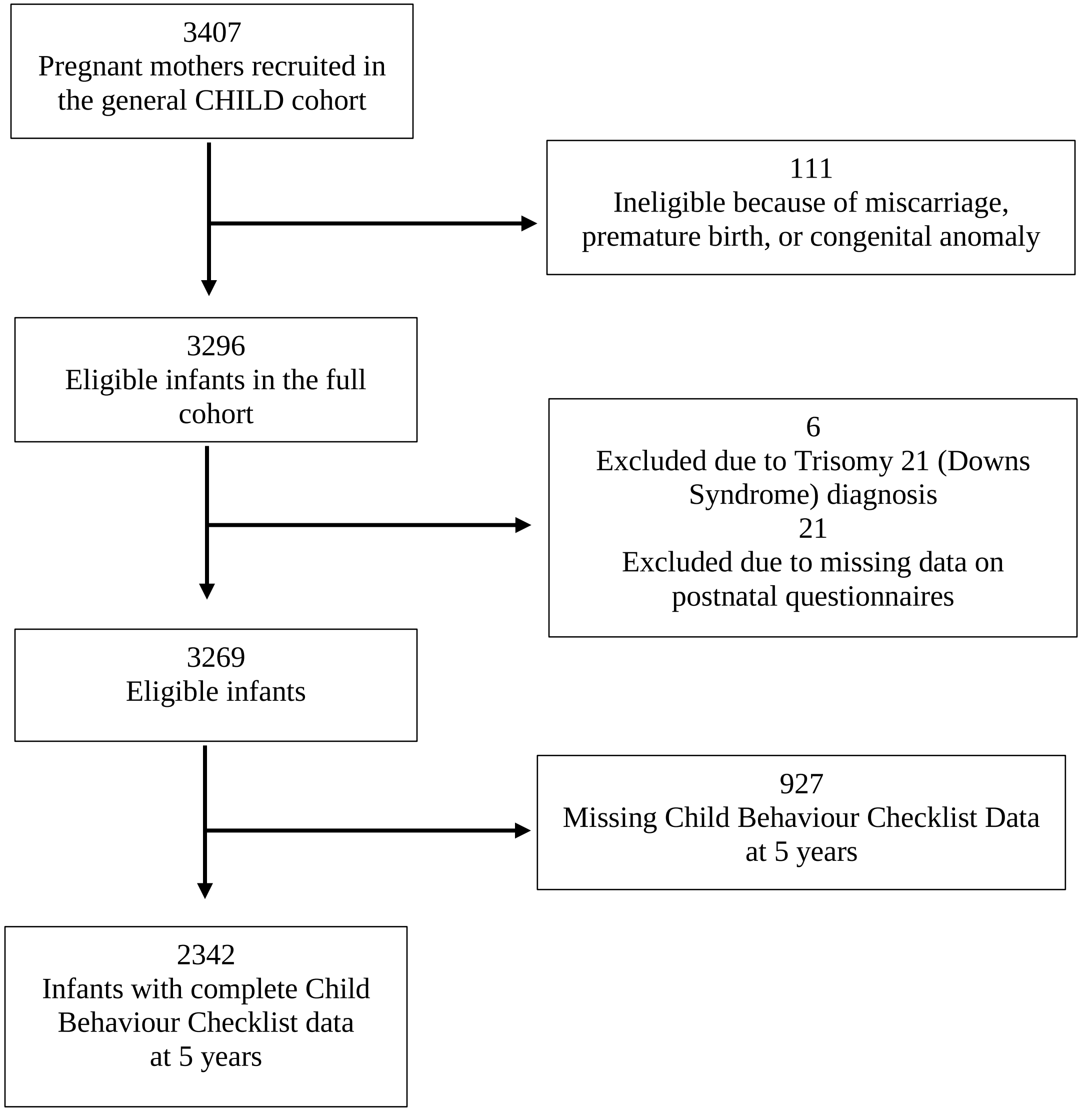

Supplementary Table 1: Unadjusted and Adjusted Regression Estimates between Breastfeeding Practices and Child Behaviour at 5 Years in the CHILD Cohort Study

Child Behaviour Checklist 5 Years	Sample n	Unadjusted β (95% CI)		Sample n	Adjusted β (95% CI)	
**Internalizing Behaviour**						
**Breastfeeding Mode at 3 Months** (reference: no breast milk)	2264			1778		
Breast milk & formula		-0.46	(-1.76, 0.84)		0.13	(-1.34, 1.60)
Breast milk only (some expressed)		**-1.64**	**(-2.88, -0.39)****		-1.27	(-2.68, 0.13)^
Breast milk only (all direct)		**-2.07**	**(-3.35, -0.80)*****		-1.37	(-2.83, 0.09)^
**Breastfeeding at 6 Months** (reference: no breastfeeding)	2305			1803		
Partial Breastfeeding		**-1.57**	**(-2.51, -0.62) *****		**-1.13**	**(-2.19, -0.07)***
Exclusive Breastfeeding		**-2.47**	**(-3.65, -1.28)*****		**-2.14**	**(-3.46, -0.81)****
**Breastfeeding at 12 Months** (reference: No)	2335			1825		
Yes		**-0.79**	**(-1.54, -0.05)***		-0.80	(-1.63, 0.03)^
**Breastfeeding at 24 Months** (reference: No)	2335			1825		
Yes		-0.73	(-2.00, 0.54)		-0.89	(-2.28, 0.51)
**Externalizing Behaviour**						
**Breastfeeding Mode at 3 Months** (reference: no breast milk)	2264			1778		
Breast milk & formula		-0.26	(-1.62, 1.10)		0.44	(-1.12, 2.01)
Breast milk only (some expressed)		**-1.60**	**(-2.90, -0.29)***		-0.60	(-2.09, 0.90)
Breast milk only (all direct)		-1.32	(-2.65, 0.02)		-0.28	(-1.83, 1.27)
**Breastfeeding at 6 Months** (reference: no breastfeeding)	2305			1803		
Partial Breastfeeding		**-1.21**	**(-2.20, -0.22)***		-0.53	(-1.67, 0.60)
Exclusive Breastfeeding		**-2.35**	**(-3.59, -1.11)*****		**-1.67**	**(-3.08, -0.25)***
**Breastfeeding at 12 Months**	2335			1825		

(reference: No)					
Yes		-0.44	(-1.22, 0.34)		-0.38	(-1.26, 0.51)
**Breastfeeding at 24 Months** (reference: No)	2335			1825		
Yes		0.15	(-1.19, 1.48)		-0.15	(-1.64, 1.34)
**Total Behaviour**						
**Breastfeeding Mode at 3 Months** (reference: no breast milk)	2264			1778		
Breast milk & formula		-0.70	(-2.00, 0.60)		-0.12	(-1.57, 1.34)
Breast milk only (some expressed)		**-2.04**	**(-3.29, -0.80)*** **		**-1.43**	**(-2.82, -0.04)***
Breast milk only (all direct)		**-2.08**	**(-3.36, -0.81)*** **		-1.25	(-2.69, 0.20)^
**Breastfeeding at 6 Months** (reference: no breastfeeding)	2305			1803		
Partial Breastfeeding		**-1.60**	**(-2.55, -0.66)*** **		-1.02	(-2.07, 0.04)^
Exclusive Breastfeeding		**-2.59**	**(-3.77, -1.40)*** **		**-2.11**	**(-3.42, -0.79)** **
**Breastfeeding at 12 Months** (reference: No)	2335			1825		
Yes		-0.65	(-1.40, 0.09)^		-0.65	(-1.47, 0.18)
**Breastfeeding at 24 Months** (reference: No)	2335			1825		
Yes		0.10	(-1.18, 1.37)		-0.20	(-1.59, 1.19)

Notes: Adjusted models are displayed in Figure 1 A of the main paper. Adjusted models in all figures and tables include the following confounders: child sex, prenatal stress, prenatal depression, study site, birth mode, birthweight, gestational age, household income, maternal education, maternal race, marital status, number of older siblings, prenatal smoking, attention deficit hyperactivity disorder genetic risk score. ^ p ≤0.1, *p ≤0.05 **p ≤0.01

Supplementary Table 2: Adjusted Linear Regression Estimates Between Postpartum Depression, Parent-Child Dysfunction and Child Behaviour at 5 Years in the CHILD Cohort Study

	**Child Behaviour Checklist Scores**		
**Variable**	Internalizing Score Adjusted β (95% CI)	Externalizing Score Adjusted β (95% CI)	Total Score Adjusted β (95% CI)
**Postpartum Depression** (Score ≥ 16)			
6 Months (n= 1599)	**2.23 (0.82, 3.63)****	**2.23 (0.74, 3.72)****	**2.26 (0.87, 3.65)****
1 Year (n=1632)	**3.37 (1.99, 4.75)*****	**3.52 (2.02, 5.01)*****	**3.71 (2.33, 5.09)*****
2 Year (n=1536)	**2.49 (1.19, 3.80)*****	**3.55 (2.15, 4.95)*****	**3.30 (2.00, 4.59)*****
**Parent-Child Dysfunction** (Score ≥ 20)			
1 Year (n=1631)	**3.25 (2.00, 4.49)*****	**3.44 (2.09, 4.80)*****	**3.49 (2.24, 4.75)*****
2 Year (n= 1542)	**4.25 (2.98, 5.52)*****	**5.07 (3.70, 6.43)*****	**5.06 (3.80, 6.31)*****

Notes: Estimates are displayed in Figure 1 B of the main paper. Adjusted models in all figures and tables include the following confounders: child sex, prenatal stress, prenatal depression, study site, birth mode, birthweight, gestational age, household income, maternal education, maternal race, marital status, number of older siblings, prenatal smoking, attention deficit hyperactivity disorder genetic risk score. Reference groups for estimates are scores <16 for depression and <20 for parent-child relationship dysfunction.
**p≤ 0.01, ***p≤0.001.

Supplementary Table 3: Unadjusted Odds Ratios Between Postpartum Depression and Parent-Child Dysfunction in the CHILD Cohort Study

	**Parent- Child Dysfunction** (score ≥ 20)	
**Variable**	1 Year<br>Odds Ratio (95% CI)	2 Years<br>Odds Ratio (95% CI)
**Postpartum Depression** (score ≥ 16)		
6 Months	**2.83 (2.04, 3.88)*** **	**3.80 (2.73, 5.25)*** **
1 Year	**4.07 (3.02, 5.48)*** **	**3.08 (2.21, 4.24)*** **
2 Year	**2.17 (1.56, 2.99)*** **	**2.97 (2.21, 3.97)*** **

Note: All estimates are unadjusted odds ratios and 95% confidence intervals. ***p≤0.001.
